# Extended Phenotype of *PEX11B* Pathogenic Variants: Ataxia, Tremor, and Dystonia Due to a Novel C.2T > G Variant

**DOI:** 10.1002/mdc3.14178

**Published:** 2024-08-02

**Authors:** Franclo Henning, Kireshnee Naidu, Christopher J. Record, Natalia Dominik, Jana Vandrovcova, Frans Lubbe, Marli Dercksen, The ICGNMD Consortium, Lindsay A. Wilson, Francois Van Der Westhuizen, Mary M. Reilly, Henry Houlden, Michael G. Hanna, Jonathan Carr

**Affiliations:** ^1^ Division of Neurology, Department of Medicine Stellenbosch University Cape Town South Africa; ^2^ Neurology Research Group, Division of Neurology, Department of Medicine University of Cape Town Cape Town South Africa; ^3^ Department of Neuromuscular Diseases, UCL Queen Square Institute of Neurology University College London London United Kingdom; ^4^ Department of Neuromuscular Diseases The National Hospital for Neurology and Neurosurgery London United Kingdom; ^5^ Neurology Private Practice Hermanus South Africa; ^6^ Faculty of Natural and Agricultural Sciences, Focus Area for Human Metabolomics North‐West University Potchefstroom South Africa

**Keywords:** peroxisomal disorders, peroxisome biogenesis disorder, *PEX11B*, dystonia, tremor, ataxia

Peroxisome biogenesis disorders (PBD) are usually the result of biallelic pathogenic variants in peroxin (*PEX*) genes, leading to generalized peroxisomal dysfunction due to the abnormal assembly or maintenance of peroxisomes.^1^ PBD 14B is a subtype of PBDs caused by biallelic pathogenic variants in PEX11Beta (*PEX11B*), encoding a peroxisomal membrane protein that is involved in the peroxisome division pathway.^1^ The first patient with a PBD caused by biallelic pathogenic variants in the *PEX11B* gene was reported in 2012,^2^ and a case series of 5 patients from 3 families were described in 2017.^3^ All patients had congenital cataracts and intellectual disability, although dysmorphism, hearing loss, and peripheral neuropathy were variably present.

A male patient of European ancestry (III‐1 in Fig. 1) with congenital cataracts and intellectual disability developed progressive sensory loss and pain in the limbs from the age of 24. At the age of 34, he developed abnormal movements in all 4 limbs and became wheelchair bound from the age of 36. On examination at the age of 43 he had a high‐amplitude postural tremor of the upper limbs with an associated rest component, and there were unusual tremulous movements in the legs, with superimposed myoclonic jerks. Marked anterocollis and dystonic posturing of the hands were also present as shown in Video 1. He had gaze‐evoked nystagmus, symmetric weakness in all limbs, lower‐limb areflexia, and a glove‐stocking pattern of sensory loss for pinprick. Limited nerve conduction studies of the upper limbs at the age of 24 showed bilateral median neuropathies, and brain magnetic resonance imaging showed generalized atrophy involving the supra‐ and infratentorial brain regions equally. His younger sister (III‐2 in Fig. 1) had congenital cataracts and cognitive decline from age 5 and developed autonomic dysfunction, hearing impairment, and epilepsy in her twenties. No tremor or ataxia was noticed by her or her parents, but on examination at the age of 41 she had gaze‐evoked nystagmus, ataxic dysarthria, limb and gait ataxia, and a mild action tremor. She also had sensorineural hearing loss, symmetric weakness in all limbs, generalized areflexia, a glove‐stocking pattern of sensory loss for pinprick, and an abnormal vibration sense and proprioception at the toes and ankles. Biochemical analysis performed in her early thirties showed a marginally increased C26:0 level and C26:0/C22:0 ratio, and normal phytanic and pristanic acid levels. Imaging and electrodiagnostic testing were not performed as she was examined at her institutional residence. The parents were nonconsanguineous and unaffected, but there was a family history of a similar condition in 2 deceased cousins (siblings, II‐6 and II‐7) on the maternal side of the family (Fig. 1).

**FIG. 1:**
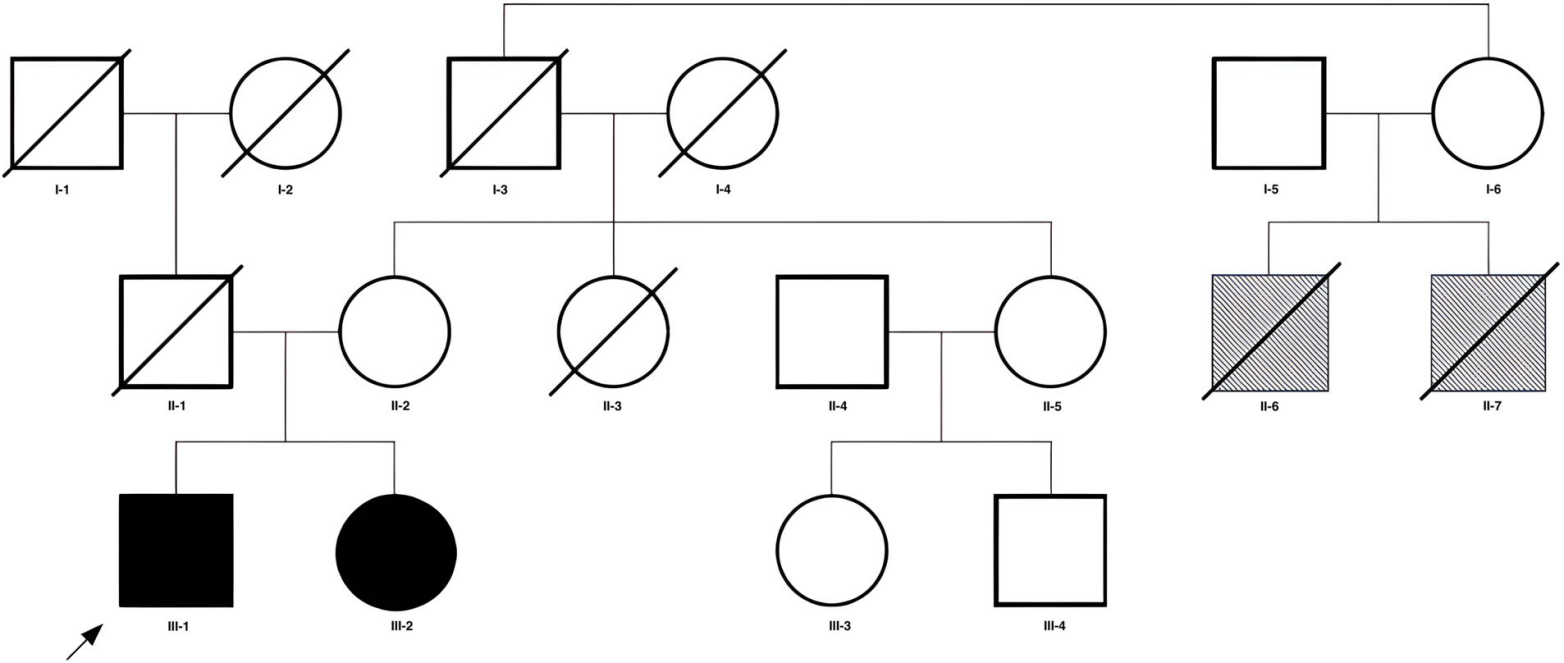
Genogram illustrating the extended family of the 2 siblings with peroxisome biogenesis disorder 14B. The proband (III‐1) is indicated by an arrow. The 2 affected brothers on the maternal side (II‐6 and II‐7, shaded) were not examined but were reported to have had bilateral congenital cataracts, intellectual disability, hearing loss, and limb weakness. The proband and his sibling (III‐2) were both homozygous for the NM_003846.3:c.2T > G (p.Met1?) variant, whereas 1 parent (mother, II‐2) was heterozygous for this variant.

**Video 1:** Video of the proband (III‐1), taken at the age of 43, illustrating postural and rest tremor of the upper limbs, tremulous movements in the legs, superimposed myoclonic jerks, dystonic posturing of the hands, and anterocollis.

Whole exome sequencing (WES) was performed by Macrogen Europe (Amsterdam, the Netherlands), and raw WES data were analyzed as previously described.^4^ Both patients were found to be homozygous for the NM_003846.3:c.2T > G (p.Met1?) variant within exon 1 (chr1.hg38:145918687A > C) of the *PEX11B* gene. Sanger sequencing confirmed the homozygous presence of this variant in both patients, whereas the mother (II‐2) was heterozygous (Fig. S1). DNA from the father (II‐1) was not available for segregation analysis (deceased).

PBDs generally present as severe neurodevelopmental disorders in childhood. However, the availability of advanced diagnostic testing, including WES, has led to the diagnosis of several patients with late‐onset PBDs, with fewer and less‐severe clinical features and no or marginal biochemical aberrations.^5^, ^6^, ^7^ Five pathogenic variants in *PEX11B* have previously been described, four of which are single‐nucleotide variants and one a deletion of exons 1 to 3.^2^, ^3^, ^8^ Based on the American College of Medical Genetics and Genomics criteria,^9^ the novel homozygous *PEX11B* variant identified in our family can be classified as likely pathogenic, as it is a start loss (null) variant in a gene where loss of function is a known mechanism of disease, and is present at extremely low frequency in population databases. In addition, this variant is in a highly conserved region, and multiple in silico prediction tools (eg, CADD version 1.7, SIFT, and PROVEAN) suggest pathogenicity. Although the distant relatives could not be examined, it is highly likely that they had the same condition.

The 2 patients described in this report presented with congenital cataracts (which appears to be a consistent early feature of the *PEX11B*‐related PBDs),^2^, ^3^ intellectual disability, and peripheral neuropathy. In addition, a complex movement disorder consisting of postural and action tremor, neck and limb dystonia, limb myoclonus, and ataxia was present in the proband, and ataxia and a mild action tremor in the sibling, a phenomenon that has not previously been described in PBDs. Although these movements may be unusual for this condition, it is also possible that the movement disorder manifests only at a later age. Indeed, whereas the oldest previously described patient with a PBD was 26 years of age,^2^ the movement disorder was noticed in our proband at the age of 34 and an asymptomatic tremor was present in the sibling at the age of 41. It would be interesting to note whether a movement disorder develops in any of the previously described cases on long‐term follow‐up.

## Author Roles

1. Research project: A. Conception, B. Organization, C. Execution/data collection/data interpretation;
2. Manuscript preparation: A. Writing of the first draft, B. Review and critique

F.H.: 1A, 1B, 1C, 2A

K.N.: 1B, 1C, 2B

C.J.R.: 1C, 2B

J.V.: 1B, 1C, 2B

N.D.: 1C, 2B

F.L.: 1C, 2B

M.D.: 1C, 2B

L.A.W.: 1B, 1C, 2B

F.W.: 1C, 2B

M.M.R.: 1B, 2B

H.H.: 1B, 1C, 2B

M.G.H.: 1B, 2B

J.C.: 1C, 2A, 2B

## Disclosures

**Ethical Compliance Statement**: This study was approved by the Health Research Ethics Committee of the Faculty of Medicine and Health Sciences, Stellenbosch University. Written informed consent for study participation was provided by the legal guardian (parent) of the patients. In addition, the legal guardian reviewed and provided written informed consent for use of the video recording. We confirm that we have read the journal's position on issues involved in ethical publication and affirm that this work is consistent with those guidelines.

**Funding Sources and Conflicts of Interest**: This work was supported by an MRC strategic award to establish the International Centre for Genomic Medicine in Neuromuscular Diseases (ICGNMD) MR/S005021/1. Fellowship for Kireshnee Naidu was funded by the Guarantors of Brain (UK Charity 1197319). The authors declare that there are no conflicts of interest relevant to this work.

**Financial Disclosures for the Previous 12 Months**: Christopher J. Record was supported by the National Institutes of Neurological Diseases and Stroke and Office of Rare Diseases (U54NS065712). Mary M. Reilly was supported by the Wellcome Trust (G104817), the National Institutes of Neurological Diseases and Stroke and Office of Rare Diseases (U54NS065712 and 1UOINS109403‐01), the Muscular Dystrophy Association (MDA510281), the Charcot Marie Tooth Association (CMTA), the Harrington Discovery Institute, and Alnylam Pharmaceuticals and Applied Therapeutics. The rest of the authors have no additional disclosures to report.

## Supporting information


**Figure S1.** Result of Sanger sequencing in the proband (III‐1, electropherogram A), the sibling (III‐2, electropherogram B), and mother (II‐2, electropherogram C) illustrating the c.2T > G start loss variant. The bottom electropherogram (D) is from an unaffected control, and the reference sequence is shown at the top.
