# Supplementary figures and images for: Extended Phenotype of PEX11B Pathogenic Variants: Ataxia, Tremor, and Dystonia Due to a Novel C.2T > G Variant
Source: Mov Disord Clin Pract. 2024 Aug 2;11(10):1298–300. doi: 10.1002/mdc3.14178 (PMC11489608; doi:10.1002/mdc3.14178)

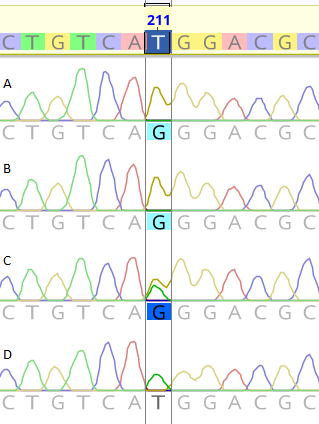

Supplement: Supplementary file 1 — Figure S1. Result of Sanger sequencing in the proband (III‐1, electropherogram A), the sibling (III‐2, electropherogram B), and mother (II‐2, electropherogram C) illustrating the c.2T > G start loss variant. The bottom electropherogram (D) is from an unaffected control, and the reference sequence is shown at the top. [file MDC3-11-1298-s001.tiff]
